# A Qualitative Inquiry of the Lived Experiences of Music Therapists Who Have Survived Cancer Who Are Working with Medical and Hospice Patients

**DOI:** 10.3389/fpsyg.2016.01840

**Published:** 2016-11-18

**Authors:** Jin Hyung Lee

**Affiliations:** Department of Music Therapy, EWHA Womans UniversitySeoul, South Korea

**Keywords:** experience of cancer, music therapist, countertransference (psychology), self-disclosure, descriptive phenomenology

## Abstract

Cancer is a debilitating illness that affects more than one in every three Americans at sometime in their life time regardless of their social, cultural, ethnic, religious, or economic status. A few studies in the psychotherapy literature have investigated the impact of cancer on the personal and professional lives of psychotherapists. However, such investigations are yet unknown in medical or music therapy literature. In this descriptive phenomenological study, the researcher interviewed five American music therapists who have survived cancer and also work with patients in medical hospitals or hospice settings. The purpose of this study was to fully describe their lived experience of surviving cancer and examine how the cancer experience affected their clinical work thereafter. The data was analyzed using an open coding method from grounded theory which identified four major themes: (a) personal significance; (b) relational significance; (c) musical significance and (d) professional significance. The descriptions provided by these participants of their cancer experience as patients, survivors, and cancer surviving therapists, have revealed various psychosocial and physical issues encountered, and numerous coping methods they employed, and poignantly explained how their clinical approach evolved and expanded due to the personal experience of cancer. Specific issues in relation to countertransference, self-disclosure, and ways of developing empathic approaches without having such personal experience were discussed in addition to suggestions for future research.

## Introduction

Cancer is a debilitating illness that affects millions of Americans every year. The American Cancer Society (2014) estimated that 13.7 million people, about 43% of all Americans have a personal history of cancer. It is the second leading cause of death in the US as approximately one in every two males, and one in every three females is likely to develop cancer in their lifetime (American Cancer Society, 2014). Fortunately, the 5 year survival rate of cancer patients has been on the rise and increased from 49% in 1977 to 68% in 2009 (American Cancer Society, 2014). What's important to consider is that cancer is not a status, class, or career disease. It impacts everyone, from bakers to homemakers, from ministers to teachers and also includes those who care for persons who are ill, such as doctors, nurses, and therapists. There are a number of anecdotal online blogs, books (Mullan, 1983; Finegan, 2004; Liberman, 2012), articles (Keoun, 1996; Morrison, 1997; Feinsilver, 1998; Tierney and McKinley, 2002; Nesbit, 2004; McCorkle, 2012), and research studies (Hott, 2000; Grefenson, 2012) discussing how cancer impacted the lives of health professionals.

One element that does not receive a lot of attention is the impact of catastrophic illness on the ability of these professionals who work with patients (Gerson, 1996). Dewald (1994) argued that a therapist's encounter with serious illness or disability can elicit major conscious and unconscious reactions in both therapist and client, and it can have either a positive or negative impact on the therapeutic process. He further discussed various countertransference issues that can arise if not addressed carefully. To further investigate this matter, I reviewed the internet postings, lecture videos, autobiographies, podcasts, journal articles, and editorials of medical practitioners who had experienced cancer (Keoun, 1996; Morrison, 1997; Feinsilver, 1998; Tierney and McKinley, 2002; Nesbit, 2004; Liberman, 2010, 2012; Coomer, 2011; Granger, 2012; McCorkle, 2012), and in these presentations, they site different coping mechanisms they employed to help them. Also a small number of scholars who themselves had cancer, conducted qualitative inquiries (Hott, 2000; Grefenson, 2012) on the impact of cancer on their clinical work as psychotherapists. Although the majority of these writings were anecdotal with only a few peer-reviewed publications, they vividly described the ways how cancer influenced the personal and professional aspects of their lives. These reviews led me to wonder if similar challenges were experienced by music therapists. This exploration was personally meaningful to me for two reasons. Firstly, I had worked with an exceptional music therapist who was a cancer survivor herself, and this experience led me to ponder upon the role of her personal experience of cancer in clinical work. Secondly, my understanding of cancer experience and my work as a music therapist, changed considerably after I lost my brother to stomach cancer and having to take care of my mother while she fought cervical and breast cancer. Consequently, I wanted to learn how music therapists experienced cancer, coped with it, and used this experience to inform their practice. In addition, I was interested in learning if and how the therapists utilized music as a way of coping with their illnesses.

## Review of literature

A thorough database search was conducted with combinations of the following keywords: health personnel, health professional, medical professional, doctor, physician, nurse, therapist; cancer, post cancer, neoplasm, illness, cancer survivor; and coping, work, practice, transformation, clinical work, experience, personal experience, countertransference using CINAHL, Medline, Proquest, and PsycINFO. In addition, a similar search was conducted on Google to locate books and online articles related to this topic. The following section summarizes the anecdotal reports and qualitative studies found in this search.

### Impact of cancer on health professionals

Many anecdotal stories illustrate how health professionals also face issues and challenges that are not very different from most people. Psychological impact of cancer often reported by medical professionals included: total shock (Tierney and McKinley, 2002; McCorkle, 2012); feelings of loss, uncertainty, doubt, depression, anxiety, and fear of making their loved ones suffer (Tierney and McKinley, 2002); and confusion over professional identity and fear of losing professional competency (Keoun, 1996; Tierney and McKinley, 2002; McCorkle, 2012). In terms of physical impact, medical professionals also expressed hardships associated with common symptoms such as pain, nausea and fatigue. Moreover, Tierney and McKinley illustrated how their fear of pain set in even before starting their treatments because they knew what to expect (2002).

Social impact of cancer was mentioned frequently as well. Tierney and McKinley noted how difficult it was for them to decide with whom to share their diagnoses (2002). They also reported feeling isolated and observed shifts in their relationships with loved ones. On the other hand, they claimed that social support was the most powerful method of coping as it provided them hope (2002). For many, it was about reaching out and connecting with others who were also fighting cancer (Tierney and McKinley, 2002; Liberman, 2010; Coomer, 2011; Granger, 2012). In fact, the reasons behind writing on-line posts, articles, and books were to help others cope. When they reached out, they learned more about others who have survived cancer, which helped them to focus on the present moment and slow down (Tierney and McKinley, 2002).

In addition to social support, medical professional utilized various coping methods which included: music (Liberman, 2012); using skills based on their professional training such as communication and counseling skills (Nesbit, 2004); and finding new meaning in cancer (Tierney and McKinley, 2002; Liberman, 2010; Granger, 2012). For instance, it was reported that cancer experiences made them reorganize life priorities, pay attention to what's important to them, keep living life fully, celebrate the value of little things in life, and treasure relationships with friends and family (Tierney and McKinley, 2002; Liberman, 2010).

As medical professionals, coming back to provide patient care was difficult as they were unsure of their ability to perform as before (Tierney and McKinley, 2002; McCorkle, 2012); they did not want the same level of intense work (Tierney and McKinley, 2002); and they became reminded of their own illness (Grefenson, 2012). On the other hand, many professionals noticed differences in their level of understanding and empathic caring for patients. Couple doctors stated that they learned how to explain medical procedures or inform test results in a more realistic and emphatic manner (Coomer, 2011; Granger, 2012); Liberman indicated how strongly she emphasizes pain management after her own experience (n.d.); and Granger indicated how she will only order the essential tests (2012). In addition, a number of the writers indicated that they became more compassionate, sensitive, empathic and caring in their work as a result of their personal experience with cancer (Alger, 1995; Liberman, 2010; Granger, 2012; Grefenson, 2012; McCorkle, 2012).

### Impact of cancer on psychotherapists

Hott (2000) interviewed 19 psychotherapists to investigate the participants' ways of coping with their diagnosis, treatment, and recovery. She analyzed the data using the coping constructs proposed by Lazarus and Folkman, which included: Confrontive coping, distancing, self-controlling, seeking social support, accepting responsibility, escape-avoidance, planful problem-solving, and positive reappraisal. In addition, she also identified ideas that elaborated on “self-disclosure.” The most frequently utilized coping strategies were: seeking social support, planful problem solving, and positive reappraisal. Occasionally adopted strategies were distancing and confrontive coping, followed by self-controlling. The least practiced strategies were accepting responsibility and escape-avoidance. In terms of self-disclosure, seven participants openly discussed their diagnosis whereas it took several months for eight therapists to disclose the news to their clients. Five participants did not share the news with their patients. Overall, the participants expressed their coping methods to be adequate, and came to a consensus that their skills as therapists were useful in their own recovery (Hott, 2000).

Grefenson's study of seven psychotherapists who survived breast cancer, focused mainly on the ways cancer influenced their clinical work, which included both positive and negative consequences (2014). All seven therapists indicated that their experience of cancer brought significant changes in their clinical work. The most frequently mentioned changes were identified as *Bringing something new to the work*. More obvious notable changes were categorized as *explicit change*, whereas more internal shifts were labeled as *implicit change*. The explicit changes in this domain included: more enhanced level of patience, compassion, empathy, empathic listening, presence, and attunement, increase in fostering growth through hardship, increased emphasis on strengths of the client, and allowing herself to be more real and vulnerable. The implicit changes encompassed increased self-awareness and self-care, feeling more comfortable as themselves, realizing their own potential to grow through hardship, acknowledging the cancer experience as an opportunity for growth and healing, employing self-care strategies to address work issues, being more accepting of the unpredictable nature of life, and moving into spiritual practice.

The changes identified as *Challenges* included: negative countertransference toward certain patients and feeling decreased patience with them, being more aware of their own illness during sessions, and having problems with memory, concentration, fatigue, and level of energy. Quite a few participants experienced *Changes in theoretical orientation* as their clinical approach became more eclectic or incorporated other modalities. As a result, they reported feeling better equipped and more able to address the various needs of their clients.

In terms of therapist's *Self-disclosure*, six participants felt they were forced to disclose their diagnosis with their patients because of prolonged absences or changes in their appearance due to cancer treatments. This resulted in deliberate contemplation over the issues of when, how, and how much to share and with whom. Most indicated that such disclosure can impact the relationship and therapeutic process both positively and negatively, while two of the participants emphasized the value of self-disclosure as it created positive momentum for a deeper therapeutic relationship. Lastly, the participants pointed out that they came to value the *Use of supervision or personal therapy* as a result of their cancer experience (Grefenson, 2012).

### Research questions

The purpose of this study was to explore the following question: (1) what is the personal impact of experience with cancer on music therapists who work in medical or hospice settings? and (2) what is the clinical impact of experience with cancer on music therapists who work in medical or hospice settings?

## Methodology

### Design

This study is a qualitative exploratory study investigating the lived experience of cancer surviving music therapists who work with medical or hospice patients. Due to the lack of information on this topic, descriptive phenomenology was used to study and describe the therapists' experiences as fully and faithfully as possible (Creswell, 2007). Descriptive phenomenology seeks to study participants' own views and meaning of their experience by describing the phenomenon under investigation with the words of the participants (Colaizzi, 1978). It promotes a better understanding of the nature or meaning of life experiences, and strives to portray the essence of that “lived experience” (Creswell, 2007).

### Participants

#### Criteria

Participants were limited to board certified music therapists working in a medical or hospice setting either on a part-time or full-time basis. Participants were also limited to cancer survivors who were in remission and not receiving cancer-related medical treatments at the time of recruitment. In addition, they were asked to confirm that they perceive themselves to be in a healthy state both physically and psychologically to participate in this study. Although the risk of participating in this study was considered to be none or minimal, I asked this question because reminiscing and talking about the lived experience of cancer can bring up intense memories from physically and psychologically challenging time.

#### Recruitment

Two types of sampling methods were used: purposive and snowball sampling. As I was planning this project, it came to my attention that there was going to be a Clinical Practice Forum entitled: “CANCER: Insights from Music Therapists who are Cancer Survivors” at the 2012 American Music Therapy Association conference. In order to meet potential participants, I participated in this forum, explained my project, and obtained contact information from three therapists who showed interests in participating.

Upon getting approval from the Institutional Review Board of Temple University, I contacted the potential participants with more specific information on the study and consent forms, and they all agreed to participate. A snowball sampling was utilized at this point to recruit more participants. I asked a few music therapists who were active in networking with other therapists nationwide, with an electronic copy of the study invitation to be forwarded to the potential participants. The email invitation also asked readers to forward the message to any other potential participants that they knew. From this process, two additional participants agreed to participate. Thus, a total of five music therapists participated in this study.

### Background of participants

Of the five participants, two therapists worked entirely in hospice settings, one solely in medical, two in a combination of medical, rehabilitation and/or hospice. All of them worked full time but one retired recently. Two of them had over 30 years of clinical experience, one had 20–30 years, and two had 10–20 years of experience. Two were over 60 years of age, one was between 50 and 60 and the other two were between the ages of 40–50. Two therapists had doctoral degrees, one had a masters, and two had bachelors or equivalency degrees. In terms of cancer, all but one had a form of breast cancer, while one suffered skin cancer. They were diagnosed with cancer between the age of 30–50, meaning it was over 20 years ago for two, between 5 and 10 years for the other two, and <5 years ago for one participant.

### Data collection and analysis

#### Interview procedure

I met with all five participants for a qualitative interview at their home or office. Upon obtaining written consents for participation and permission to audio-record, a semi-structured interview was conducted with each individual which lasted approximately 70 min. Initially, interviews began with a general question about their clinical work and background, then more focused questions about their experience of surviving cancer and working with patients followed. In order to capture the most vivid and subjective experience of these therapists, I asked all questions in an open-ended manner and adapted questions to obtain more individually relevant and meaningful issues. A list of the main interview questions is attached in Appendix A. During the interview, I mainly utilized attentive listening and reflecting, and probing and clarification when necessary to capture the experience in a more detailed and vivid form, or to clear up any chance for misinterpretation due to nuances or metaphoric use of language.

#### Reflective log

After each interview, I kept a reflective log on my thoughts, feelings and observations for the purpose of keeping my stance as a researcher in check. Also, I used these logs to further inform the data analysis process.

#### Researcher's personal stance

In addition to keeping in mind my personal relationship with this topic as a person and a therapist through the reflective logs, it was necessary to examine my own assumptions on the role of personal experience in therapy practice. I don't assume that having a personal experience of illness indicates automatic improvement in one's ability to practice as a therapist. However, I believe that illness provides opportunities for personal growth, better comprehension of the influence of such illness, and increased confidence in coping methods one has utilized for oneself. Thus, I feel that there is a significant role that personal experience of illness plays in one's clinical practice. My assumptions for this study therefore are: personal experience of illness has a positive clinical impact on therapists; personal experience of illness influences therapists' ability to understand their patients; personal experience of illness provides therapists with more opportunities to grow as a person; and personal experience of illness influences their relationship with music, especially with the therapeutic value of music.

#### Process of data analysis

I analyzed the data by first reading each transcript until I got the essence of what the interviewees presented. Then I used open coding techniques from the grounded theory data analysis steps illustrated by Corbin and Strauss (2008). Unlike proceeding studies that focused only on coping or clinical impact of cancer on psychotherapists, the scope of this study was much broader encompassing the experience of cancer surviving music therapists all the way from diagnosis and coping with illness, to working again as therapists. This is a phenomenologically-oriented study that borrowed data analysis methodology from grounded theory, but was not intended to develop a theory. This methodology is considered a “modified form.” As Charmaz noted many studies utilize grounded theory to “develop rich conceptual analyses of lived experience” (Charmaz, 1996, p. 48). The purpose of using the open coding technique from grounded theory was to systematically generate data by employing techniques suggested by Corbin and Strauss (2008) and to gain new insights into the multilayered meanings of coping and working through cancer. To assist the process of constant comparison, categorization, and repeated editing, a qualitative data analysis software called MAXQDA11™ was used. The steps in data analysis were as follows:

1. I transcribed each interview verbatim from mp3 audio files.
2. I eliminated unnecessary or repeated words such as “I know, um, I mean, etc.” Also, I deleted all identifying information such as names and locations from each transcript.
3. I sent all transcripts back to each participant, asked them to check the content, invited them to modify or elaborate further if they were not satisfied with their original statement, and when necessary, requested clarification on certain points that were ambiguous.
4. Upon confirming the content with each participant, I read each interview transcript at least twice in its entirety to get a sense of the whole.
5. During the third reading, a concept of each unit was identified and assigned with a new code through the inductive method of coding. As it progressed, all concepts were consistently compared to each other to determine if a new code was needed or if it could be labeled with previous codes.
6. Initially, descriptive codes were considered to capture the essence of the experience.
7. All codes were reviewed and examined to discover codes that have similarities between them or share similar properties in order to form categories. Steps 5 and 6 were repeated to develop categories that better represented the data.
8. Categories were examined to form major themes.
9. An independent research advisor reviewed the transcripts and coding system created from steps 5, 6, 7 as a means of triangulation. Based on this process, steps 6 to 8 were repeated to refine the coding structure and analysis of data.

#### Integrity and trustworthiness

I wrote reflective logs after each interview in order to gain awareness of my thoughts, feelings and assumptions in relation to this topic. Participants were asked to review the transcripts in order to check for accuracy and completeness of the data for the purpose of *member checking*. If there were inaccuracies, they were asked to edit or further elaborate on their answers if desired. Upon establishing initial codes, themes, and code structures, all materials were reviewed by an independent research advisor as a method of *triangulation*. This step was repeated many more times until agreement was reached on the coding process. The research advisor and I decided not to proceed with member checking on the completed codes and themes generated, which is usually carried out to secure *confirmability* in other qualitative studies. The reason for this decision was because phenomenology does not seek one truth that can be confirmed by participants. Morse et al. (2002) supported this idea by suggesting that *confirmability* may not be suitable in phenomenology because reality in phenomenology is “dynamic and changing.” Throughout these different phases, I maintained a journal reflecting on my thoughts, assumptions and feelings especially during coding and data analysis as a means of examining my pre-conceptions.

## Results

### Emergent themes

Based on the inductive analysis of the data, four major themes have emerged: (a) personal significance; (b) relational significance; (c) musical significance and (d) professional significance. These themes were derived from the main coping mechanisms utilized by the participants throughout their course of recovery and returning to work as therapists. Each of these themes was important in helping the participant form her identity as a cancer surviving music therapist. Table 1 shows the overall number of codes derived from the analysis.

**Table 1:** **Overall coding frequency by participants**.

**Themes**	**Frequency by participants**					**Total**
	**A**	**B**	**C**	**D**	**E**	
Personal significance	7	7	23	16	17	70
Relational significance	8	7	17	14	6	52
Musical significance	12	4	3	8	6	33
Professional significance	49	19	27	12	28	135
Total	76	37	70	50	57	290

### Personal significance

“I'm still learning from that experience. I'm not finished. I'm still surviving, still a cancer survivor.” This statement by one of the participants illuminates the personal significance of surviving cancer. Some of the personal processes participants encountered during their coping with and surviving cancer included: intrapersonal processes; and self-care and therapeutic experiences. These various personal processes that they have experienced and are still facing, illustrate how their lives as a person and a therapist continue to get affected by their experience of cancer.

#### Intrapersonal processes

##### Denial

Denial was a frequently utilized coping method. Initially, denial was used to postpone overwhelmingly intense emotional reactions as one participant commented “Okay, they've seen stuff before, so I'm not going to get too upset…Deep down inside I knew, but I really didn't want to admit it.” Another participant noted “I'm not sure that I wanted to completely get all mushy about it.” Other times, it was used to convince themselves that it was not bad, “I remember saying to somebody that I was having the best summer I'd ever had. Well, it wasn't.” For one participant, it was a way of focusing on the client: “I actually feel like I did great work during that time…I kept pushing the thoughts about my diagnosis away.”

##### Accepting the feelings and facts

Rather than denying what was happening, sometimes it was important to just accept the feelings that were rising. As one recalled, it was essential to admit that “sometimes I'm going to feel awful, sometimes I'm going to fail.”

##### Remaining positive

One of the two ways of staying positive was to utilize self-affirming messages to cope. One participant told herself “You'll get through it, and you will get passed it,” and another said “Okay I can do this, I can get through it and I did!” When discouraged by not being able to function as before, one participant responded “Oh boohoo, there are plenty of other things you can do.”

In addition to positive self-talk, participants also sought to find things to help them remain positive and hopeful. One participant described this as “I was trying to be thankful for every moment, and trying to help my patients also make the most of every moment.” On the same note, another participant stressed how she learned “to treasure every moment.”

##### Expansion of self-awareness

All participants mentioned self-awareness as the key intrapersonal process in coping with their cancer and in getting back to work as a music therapist. Helpful self-awareness steps included “being aware of own feelings…(including) feelings about death and dying,” “going through the stages…and understand how you are, where you fit, and if it's okay,” “(being) honest with myself,” and “being aware of what you can do, who you can and cannot work with.” In order to promote self-awareness, it was important to “have ears to hear feedback” and “keep ourselves [and] remain teachable and open…(being) open to growing.”

##### Maintaining spiritual faith

Two participants emphasized how important it was for them to hold onto their spiritual faith during their coping. One participant recalled the spiritual message she and her husband held onto: “We believed this for as long as we can remember and now we might be put to the test. This is no time to think differently. That's the way it's going to be, you believe it, I believe it. Let's live like it.” For another participant, this spiritual faith in God led to having “faith in the surgeon, doctors, treatment team, and in the treatment itself” as “have faith” was the ingrained message she got from her mother.

##### Finding meaning in cancer

Reflecting on the meaning of cancer, one participant learned to live life as “it is a marathon, not a sprint…progressing one step at a time.” It also helped her to rediscover the value of everyday life as she continued “I gained the most…when I struggled the most. To come out of that and to be able to experience those happier times…I call it in-my-life again…Cancer changed my whole life.” In the same way, another participant indicated that cancer “brought so much more knowledge” and helped to gain “more appreciation for what the patients and families went through.” She concluded “cancer made me a better therapist…it was one of the reasons I had to have it.”

#### Implementing self-care strategies

Participants talked about utilizing self-care strategies and therapeutic experiences. One strategy was gainning more information about their illness and treatment process. This meant utilizing resources that were online and offline, and also consulting with other people. Having a sense of what was going on with their own bodies and treatment was important as it meant regaining control and autonomy.

Another method was developing necessary self-care strategies to prevent future diagnoses or development of cancer. These included continued routine medical check-ups and self-examinations for everyone. In addition, one participant sought counseling as she reported “I needed the support, I needed the ideas of how to cope and how to help my daughter cope.”

### Relational significance

#### As a cancer survivor

Several participants stressed the power of support from people around. One participant said: “We have a need to connect with people for a reason. You don't ever have to walk through this by yourself.” Another participant noted how revealing her diagnosis sparked supporters: “I definitely think sharing affects how we cope with it…Getting support from others, knowing others cared…That's what helped me cope.” Revealing their diagnoses helped them to receive support from family, friends, fellow patients or cancer survivors, and professionals.

##### Support from family

The most significant source of support came from their family. One participant reminisced: “My family made me a prayer blanket…As each one tied a knot, they said a prayer or wish for me.” Likewise, family members accompanied the participants throughout the diagnosis, treatment, and recovery process, often physically, if that was not possible psychologically and spiritually. “Love helped me cope and feel supported. Love from family, friends, and God” said one participant. A message from her mother before passing, supported one participant throughout the process to the present moment as she sad: “The day of my biopsy my mother gave me a little bear that said “Have faith” and it became my symbol.”

##### Support from friends

Participants shared various ways they received support from their friends. Sometimes friends were there to talk and listen, bring food and gifts, donate their talent in taking care of the participant, or share ideas on coping strategies. One friend, who was also a music therapist, came with her guitar and provided procedural support with vocal/song improvisation which the participant recalled and said “(The procedure) was gross…so awful…Then she was improvising on a Celtic tune…my ears were happy with whatever she played on it, and before I knew it, they were done with it (procedure), and I felt so much better!” Another participant described her experience with a friend who gave her a memorable gift: “A woman from my church gave me a see-through negligee as a gift after I had been diagnosed and she knew that my breasts had been removed…She looked at me and said you are still beautiful! And I will never forget that!”

##### Sharing support with other survivors

“That's where I grew the most,” said one participant who shared her powerful experience of being part of a cancer support group. She explained further by saying “They were people just like me, going through the same thing, I learned that I wasn't the only one who thought like that or who felt like crumbling.” On the other hand, another participant noted not getting the support she needed from her support group: “They were just into what kind of wigs they were getting, and I felt they were very focused on their illness and what might happen, and I didn't want to think about that.”

One of the participants remembered calling a friend, who was a cancer survivor, and noted how helpful that friend was. She said “she talked me down off the roof.” “She asked me what stage, and I told her it was stage I. And she's like “Oh girl! Mine was stage III, you are going to be fine!” So that helped to hear and then she talked me through a bunch of things and calmed me down.”

One participant mentioned “a sense of connection” she felt with other cancer survivors. This connection was one of the reasons why they were motivated to support other survivors, speak, and present on cancer and music therapy, advocate for women's health and routine medical check-ups, and participate in this study. She illustrated: “I don't care who knows if it can help someone else or provide support and comfort.” Another participant stressed “Ladies, get your mammograms!”

##### Need to trust the doctor

One of the relational challenges participants experienced was the encounter with medical staff. These encounters created feelings of mistrust and resulted in getting a second opinion or switching to a new doctor all together for three of the five participants. Recalling this, one participant noted “I wanted a doctor who showed compassion and understanding.” Getting a second opinion was also another way of making sure that they were making the right medical decision and one participant said “I went to see an oncologist, before I had the mastectomy just to make sure that was what she thought should happen too because I figured the surgeon is always going to say “Let's cut things off” you know.”

### As a professional

The participants encountered situations that required extra attention on boundary issues with patients. They noted that these issues often called for mentorship or supervision.

#### Maintaining a healthy boundary

Participants recounted events that made them concentrate on maintaining healthy boundaries with their patients otherwise they felt “distanced,” “guilty,” or self-possessed. Thus, they intentionally separated their issues from their patients' as one said “Get out of the way, it's not about me at all” and another said “They are my patients. I love them but I love them differently from the way I love my friends and family.” A participant referred to it as “click in to music therapist mode.”

#### Sensitivity toward self-disclosure

Four of the five participants had revealed that they were cancer survivors to their clients, but everyone echoed that they only share when they were absolutely sure that it was for the “patients' benefit.” One participant elaborated on this by saying:

(I only share when) “I was led to.” If there was a certain something I heard that person say that made me think, they're not listening because they don't think I understand. If I sense that maybe, if I say I've walked in those shoes too…they listen with different ears…(Knowing it's) going to be helpful to that person…takes discernment. I don't want to wear it like a badge, like entitlement…If I'm sensitive enough to know when to open up and say that…I've seen where that gives an added assurance or confidence to them, then it's fine.

Another participant also described an outcome to disclosing as, “patients have actually had a really good reaction to finding out that I did know in that way.” It also provided a “connection” for some patients. On the other hand, everyone stressed that self-disclosure can be harmful when it is “irrelevant or self-serving,” or if it is presented in an “insensitive manner.” One very important thing to note was that “there are always things that are going to be different about the other person's experience, so I don't want to act as though I know what you're going through, because it is different.”

#### Valuing mentorship

Several participants recommended having a colleague or mentor to work through difficult cases or challenging situations. One participant noted: “You should be aware that you are an infinitely creative person (with) infinite ways of looking at things…And if you can't, find somebody who can help you look at [things in] different ways.” Another benefit of mentorship was checking-in with oneself and preventing situations that lead to negative countertransference. She described: “We always go bounce everything off each other or if it was a patient she couldn't handle, she would have me go and vice versa.”

### Musical significance

Music played a significant role as part of the participants' own coping methods. Participants also gained new insights about the therapeutic power of music through their personal experience of using music for healing.

#### Enhanced relationship with music

One participant reported using music to promote physical relaxation during her chemotherapy. For another participant, music and imagery provided procedural support during her mammogram and radiation therapy. She said “I listened to the music the whole time, and that's the only thing that got me through it.” Then, she further illustrated how she even incorporated the sounds of the machine as part of music and images during radiation therapy. She also added that she continued to listen to music while waiting for her surgery in the pre-op holding area which helped her to manage her anxiety, and further commented “To me, it was all about the music.”

For another participant, playing the piano was a vehicle for emotional expression as she said “I actually played more myself rather than listening to it. It was my own outlet…” Similarly, one participant stated, “I was able to express and experience emotions through the arts.” Lastly, one participant explained how she composed an original song to send a positive and supportive message to herself and fellow cancer survivors.

#### Trusting the power of music

Based on their own experiences with music both as patients and therapists, the participants gained a new level of trust in the therapeutic power of music. After experiencing music therapy as a patient, a participant enthusiastically stated: “I realized, really know that it's very effective…There was just a little piece of me that it might be a coincidence…But no, it is the music!” Consequently, she stated “[this experience] made me very intentional about the music I use.” “I use non-verbal music a lot more than I used to…It takes energy to process speech. And when someone is actively dying, I use music that's familiar to them, that's comforting to them…I want them to, just be surrounded by it.”

Another participant expressed having this realization of the power of music, as she stated: “I wasn't sure exactly the mechanism that did this, but what I was sure of, is that it had helped me. It helped me finally to fall asleep at night. It helped me to express what was going on inside that I couldn't…”

### Professional significance

Participants were asked to reflect on how it was for them to see patients again, and what they value in their clinical work now that they have an added perspective as a cancer survivor.

#### Awareness of countertransference issues

Returning to work with patients during or after their own cancer treatment brought certain concerns to the participants. One participant recalled feeling “afraid of going back to work” because she did not think she had “enough energy to connect with patients or fully embrace them.” Consequently, she revealed feeling “a little tentative” about seeing patients. Another participant remembered anticipating her potential new job at a pediatric cancer center to be “pretty heavy” as she said it might “touch off things in me that I did go through, didn't quite go through, might have gone through that would've come up for me in a more emotional way.”

Another emotional reaction was feelings of relief and gratitude. Seeing patients again made participants think “this could have been me,” and “I was really lucky.” There were also feelings of guilt as one participant stressed “I really felt like I was a little distanced from everybody. I didn't feel like I was a terribly effective therapist.” Seeing people or patients dying from breast cancer made one participant wonder “How did they allow it to get to that point … A lot of them just didn't take the precautions that I was very fortunate to have.”

#### Shifts in clinical emphasis

The participants had a wide array of ideas and strategies on effective ways of helping patients. Among them, the most frequently mentioned idea was “client centeredness.” Although four of the five participants indicated having been trained in the behavioral/cognitive/medical model, they considered their practice to be “humanistic,” “client-centered,” and/or “Rogerian.”

##### Client-centeredness

For the participants, the client-centered approach encompassed much more than just identifying with patients empathically, understanding patients' needs and providing individualized approach. The unique side of their take on client-centeredness was that these therapists were better informed at greater depths and levels, by being mindful of how it was for them as patients. Many participants echoed “I know what it's like to go through that.” One participant said “(Previously) I totally had a different understanding of everything they went through.” Hence, this deeper level of understanding helped them better articulate therapeutic strategies and ideas.

*Considering different phases*. Two participants emphasized the importance of knowing how illness progresses and affects patients differently. Knowing this can help the therapist better understand patients' needs and provide adequate help. One participant explained “the difficulties in the beginning of first being diagnosed versus starting treatment versus 5 years out of treatment are very different.”

*Knowing what is important in the moment*. One participant shared a story to indicate how she is better able to offer what each patient needs at each given moment. The essential part of our work is “finding what's important in the moment, what we can do, and doing it.” She then warned that providing help without knowing clients' needs are, can be a “smack of disrespect in a time that's so poignant.” Consequently, she advised, we need to “learn how to wait for the right moment, learn how to take that step when the door opens, and know sometimes there's nothing I'm going to be able to do.”

*Need to be heard*. Many participants emphasized the idea of “talk less and listen more.” They expressed this by saying “I am left reticent with my patients,” “Being silent can be as good of a gift as song,” and “They are being heard, listened to, and they're given the opportunity to be open.” Listening is considered to be a quick and valuable method of assessment as two participants noted how much they learn about each patient by listening.

*Reinstating dignity*. Some participants recalled having degrading or humiliating experiences during their battle with cancer, and emphasized the importance of reinstating dignity and empowering patients. One participant specifically noted that “it's important that they don't lose themselves in their disease. They need to be embraced for who they are, not just what they have, it's not just their disease.” Another participant reiterated this as “finding the need to assert your humanness, where you are not just a big pile of disease. We can find a way to give you a life back, maintain your life with some grace, dignity and joy.”

*Care and affection*. Client-centeredness also included providing genuine and loving care. One participant expressed her frustration as some of her colleagues at work treated patients like objects: “Don't you care who they are? They are all different…” She continued: “I find something to love about them when I first meet everybody…because when you love someone, you are more interested in them…You will be willing to take the effort and time to find out what it is that makes them do well.”

##### Holistic approach

One participant noted “Sure we can help them with their pain management through neurologic music therapy…We can do those things. But they need healing in other ways.” She added “I can weigh (different needs), so that I can address the whole person not just the physical side…It's getting a balance for that person, who may not explain that on their own without a little bit of guidance.” Another participant also made a similar remark as she said: “I'm strong on looking at the whole person, physical, emotional, spiritual, looking at everything and including the family in that.”

##### Therapeutic intention

As an extension to genuine caring, one participant presented this idea of therapeutic intention. She asserted: “The entrainment that we do…intention has more to do with it than anything else.” She was convinced that the therapeutic intent of the therapist is behind all therapeutic process. She further noted “It is…your being, your loving presence, and don't think for a second that she (unresponsive patient) is not aware of that! They know you are there.”

##### Promoting expression

Having opportunities for self-expression was considered to be an essential part of the healing process by many. One participant anticipated: “Music therapy is really most useful for helping people experience and express their emotions.” When music facilitates this expression, “they are being given a voice” and do not feel as “afraid to talk about it (their concerns).” One participant said “I used music to create a song, to write lyrics, and improvise…I was able to put it out in front of me in a way that I could share with other people.”

##### Flexibility

Addressing clients' individual needs in a timely manner requires much flexibility on music therapists' part. One participant indicated that the most important quality of an effective therapist was “flexibility…Just in being able to enter the situation, and focus on what the patient needs right away.” It's about utilizing what we have to the fullest, as asserted by another participant, “You have to be able to find a way to use what you are bringing to the party the best way.”

Being able to adapt and utilize music spontaneously was also a big part of their practice. One participant stressed “Live is better than recorded because you can make those very subtle changes in tempo and intensity of your playing while you are watching them.” All in all, “you should be aware that you are an infinitely creative person. You have infinite ways to look at things. Infinite!”

##### Eclectic practice

This extended flexibility led one participant to become “not to be so stuck in one model.” She further stated: “To me, it's about what is going to be most effective for that particular patient at that particular moment in time.” Another participant affirmed this by saying: “I don't know that there's any one approach that I would choose…It's about the patients.”

##### Relational support

The participants repeatedly brought up the importance of bridging gaps between patients and family, family and medical staff, and also between patients and medical staff. One participant said that she was often asked to go in to “encourage the family to be part of the process” and also mediate when tensions arise between all involved individuals including medical staff.

One participant paid more attention to providing the necessary support doctors are not able to provide when giving bad news to patients. She stated: “Doctors speak and speak, but patients don't know what they say…So, if the doctor comes in, I would always try to stay, even more if I know they are coming in to give bad news because I know they would drop the bad news and walk out and leave the patient devastated.” On the other hand, it was also important to pay extra attention encouraging patients to trust their doctors, as another therapist asserted: “It's important to have confidence, trust and rapport with your doctor…That's one thing that they shouldn't have to deal with, they shouldn't have to be frightened about their doctor.” In addition, another participant disclosed that she tries to nourish the caring heart among other medical professionals, as she stated: “I try to get the team to understand that there is a person there who responds well and [can] bloom in this environment. If I can share that with my team, that person is going to have a much better experience.”

##### Trusting musical space

Music provides “a safe place for patients,” and being in this space “helps them feel better.” “Using music to get there, is so much easier than verbal processing cause you know you've got that soft roundedness (in) music.” One participant highlighted this effectiveness of musical space further by commenting: “(The) music environment doesn't touch them but it holds them, and it's amazing how patients can relax and rest into it.” At times, she provides this musical space for patients who are “actively dying but they are so restless they can't let go.” Then she creates this “nice holding environment with music and makes the music what they respond to.” Although they are not responsive, she says “you can tell they like it.” In times like these, she adds “I really feel that music is really useful”

#### Professional growth

Almost everyone considered their personal experience of surviving cancer to have a positive influence on their professional work. One participant claimed “I do know that I am a more effective therapist than I was before,” and another participant said “cancer made me a better therapist.” The other participant felt the growth was ongoing: “I thought I was pretty good before that, but I think I am growing. It keeps growing and growing.”

##### Enhanced confidence

One of the signs of professional growth was feeling more confident. One of the participants indicated “I am better equipped for what happens.” Another noted “When I'm with a patient, I'm more comfortable in trusting that I'm seeing their needs…More accepting, instead of this need to fix.” This was echoed by another participant who said “I'm more comfortable knowing that that is enough, and just going with whatever appears to be needed and not questioning myself.”

##### Taking risks for patients

Participants also disclosed feeling confident in trying new things and taking risks. One participant shared: “I gave myself a little more permission to go there with her.” Now, she says “I think it allows you to go further” and “push a little more…just to see if they really don't want to be bothered.” “I am braver about suggesting things than I was certainly five 6 years ago.” She concludes, “If you feel like you are strapping on your sword and armor for your patients, you will find many more ways to care for them…and have extra ways to connect with them because you've been where they are to a degree.”

## Discussion

Five music therapists have shared their experiences of surviving cancer and returning to work as therapists. As survivors, cancer had a significant impact on their personal, relational and musical dimensions of self. The personal self was touched by powerful intrapersonal journeys through denial, acceptance, optimism, self-awareness, spiritual faith, and/or discovery of multiple meanings in cancer, in addition to their own self-care and therapeutic experiences. The relational self as patients, was affected by the incredible love from family, unexpected and humbling support from friends, deeply-felt connections with other patients and survivors, and/or compassionate and trustworthy medical care staff. As a professional, they learned to value healthy boundaries, self-disclosure, and mentorship. The cancer experience brought changes in their relationship with music as well. As patients, they witnessed the therapeutic effect of music to provide deep relaxation, powerful imagery, creative outlets, therapeutic self-expression, and effective procedural support. As music therapists, they gained even higher levels of trust and respect for music, which fostered more direct and intentional use of music.

The cancer experience also impacted the professional dimension of self in terms of countertransference, clinical emphasis, and professional identity. As they returned to clinical work, participants encountered countertransference issues of inadequacy, low energy level, unavailability, feelings of guilt, discovery of unconscious issues, identification with patients, and/or intense emotional reactions. On the other hand, they also experienced a heightened capacity to understand clients, empathize with them at a deeper level, and utilize client-centered approaches on a fuller scale. In addition, they recognized increased flexibility and freedom in music, method and even therapeutic orientation. Furthermore, several came to value their role as a bridge between patient and family, and patient and medical staff. Lastly, the therapists came to see themselves as more confident and effective therapists who trusted their work and felt comfortable taking necessary risks for their clients.

### Study findings in relation to reviewed literature

#### Challenges

In the literature reviewed, medical professionals reported realizing the importance of emotional care from their personal experience of cancer (Granger, 2012) while music therapists recognized the value of holistic and patient-centered care. At the basic level, they are similar in that both views center on the idea of holistic care. The difference then, is the scope and degree of patient centered care as psychosocial care has always been a big part of music therapy practice.

Medical professionals, doctors in particular, reported suffering from anticipatory pain prior to their cancer treatment (Tierney and McKinley, 2002) which was not reported by the music therapists. The reason seems to be obvious as music therapists do not have the same level of understanding or exposure to the details of how chemical agents work or elicit pain responses.

As clinicians, both professionals expressed concern about diminished capacity to perform their job. One difference was that medical professionals were concerned with their concentration and memory in particular (Tierney and McKinley, 2002), whereas music therapists noted being worried about their level of energy and availability in addition to concentration. The main difference between the two was the placement of emphasis when considering their jobs, memory vs. availability, or stated differently, cognitive capacity vs. empathic presence. This seemed to be the result of differences in focus required of these professionals.

When returning to work, both groups reported experiencing difficulties. One nurse reported having intense symptoms similar to that of PTSD (McCorkle, 2012) while music therapists did not report any experience of that type. One music therapist did note having an alarming response when she saw a hospice patient who shared a similar diagnosis with her, however she did not perceive it as intensely as the nurse reporting PTSD responses. This may be due to the differences in training in that music therapists are trained to address issues in the psychological domain, and utilize advanced music therapy techniques and resources for their own recovery, which in fact was true for most participants. Certainly, it depends on the specific medical setting in which one works, but another explanation could be due to the differences in number of medically urgent and frail patients that medical professionals see.

#### Coping methods

There were remarkable similarities between the ways these professionals coped with cancer. The only distinguishing factor between the groups was the use of music. Although one of the doctors emphasized the importance of music in her recovery (Liberman, 2010), she only utilized the receptive methods while music therapists used compositional, receptive, and recreative methods in conjunction with relaxation and imagery techniques. One music therapist even received a music therapy session during a medical procedure.

It would be interesting to compare the prevalence of specific coping mechanisms utilized by cancer surviving music therapists and psychotherapists based on Hott's method of analysis (Hott, 2000). However, this comparison is not possible as the scope of topic and types of questions asked were immensely different. When looking at the major coping strategies discussed so far, I can see the similarities in that both groups relied heavily on social support and positive reappraisal, which in this study was coded as “finding meaning in cancer.”

#### Clinical impact

All involved medical professionals, psychotherapists, and music therapists viewed their cancer experience as having a positive impact on their clinical practice. However, music therapists experienced professional development that was different from everyone else. This development involved forming different relationships with music, nourishing higher levels of trust in their therapeutic medium, benefitting from their own therapeutic techniques, and developing more direct and intentional styles of applying music in their practice.

Due to major differences in clinical settings and needs of the patients, there were differences found in the types of countertransference issues encountered. Because the music therapists worked in medical hospitals or hospice settings, they worked intensely with many cancer patients or hospice patients who were fighting or dying from cancer. This created unique issues such as identification and having feelings of guilt as one participant said “this could've been me.” However, there appears to be another reason why they work more effectively as one therapist said “I really know now what it's like.” On the other hand, psychotherapists had issues that were on the opposite end of the spectrum. Because their work was rarely with persons with medical problems, psychotherapists reported getting impatient with clients who overreact to things that were trivial, or not as terrible as they seem (Grefenson, 2012).

Both psychotherapists and music therapists reported shifts in their approach in treating clients. They were more eclectic and inclusive of other modalities and approaches in their work, and found this inclusiveness to be more helpful in addressing the various needs of their clients. In addition, both groups valued the importance of self-care and professional development through supervision and mentorship.

One last clinical impact of cancer on music therapists that was different from others was how the experience led them to take a role in bridging the gaps between patients and family members, and patients and medical staff. They were often present after patients received a poor prognosis, offering music and themselves in support of the news patients had just received. Additionally, they were able to help patients inform family members, and help family members better understand how to support each other. The music therapists were also able to help the patient see the doctor in a more human way, and occasionally, help the doctor see the patient differently as well.

#### Self-disclosure

The ramification of self-disclosure turned out to be quite different for music therapists and psychotherapists. The general consensus was that self-disclosure is a highly sensitive matter that therapists should be careful of. However, the biggest difference was that music therapists consider it as a means to enhancing the therapeutic relationship or trust, whereas psychotherapists consider it as a part of ethical standards, meaning that there was a compulsory factor to their self-disclosure.

Interestingly, there was a split among music therapists as well. Three of the music therapists who saw medical patients, self-disclosed their cancer experience to certain patients and felt good about it as long as it was for the right reasons, but the two remaining therapists who worked only with hospice patients never shared with their patients and said “(I would share) If it could help them…So many of my patients are cognitively impaired…” and another participant explained “I have not, maybe because the quality or the experience that I went through was relatively minor compared to what a cancer patient in hospice (goes through).” It would take more detailed and targeted interviews in order to investigate this issue further, but perhaps this disparity is due to the differences in prognosis of patients. Therapists are mindful of which stages their patients are in physically and psychologically, and also pay attention to which combination can best help them to overcome the current challenges that they have. Sometimes, patients need to remain hopeful and courageous and other times they need to find ways to gain peace and perspective over their situation and move on, and perhaps these stages are related to how the therapists decide when to self-disclose and when not to.

### Implication of cancer experience for music therapists

Table 2 shows a more detailed information on the frequency of codes mentioned by each participants. Codes that were most frequently communicated include: “Client centeredness,” “Awareness of countertransference,” “Self-awareness,” “Denial,” followed by “musical significance.” Considering that the “Awareness of countertransference” was addressed following a focused question that I asked, it can be summarized that the participants had most to say about developing a client-centered approach with deeper level of self-awareness and enhanced trust for music, despite their initial use of denial as a coping mechanism. This is meaningful in that these frequently mentioned topics reflect how the participants answered the question: “How did your cancer experience impacted your clinical work?”

**Table 2:** **Detailed coding frequency by participants**.

**Themes/Categories/Codes**	**Frequency by participants**					**Total frequency**	**Number of contributors**
	**A**	**B**	**C**	**D**	**E**		
**PERSONAL SIGNIFICANCE**							
** Intrapersonal Processes**							
Denial	1	1	1	5	12	20	5
Accepting the feelings and facts	1					1	1
Remaining positive		2	1	2		5	3
Expansion of self-awareness	3	1	8	4	5	21	5
Maintaining spiritual faith			5	2		7	2
Finding meaning in cancer			8	1		9	2
Implementing self-care strategies	2	3		2		7	3
Subtotal	7	7	23	16	17	70	
**RELATIONAL SIGNIFICANCE**							
**As Survivor**							
Support from family	1	2	2	5		10	4
Support from friends		1	1	5		7	3
Sharing support with other survivors			4		2	6	2
Need to trust the doctor	2		1	2		5	3
**As Professional**							
Maintaining a healthy boundary	2	2	4			8	3
Sensitivity toward self-disclosure^*^	2	2	4	1	4	13	5
Valuing mentorship	1		1	1		3	3
Subtotal	8	7	17	14	6	52	
**MUSICAL SIGNIFICANCE**							
Enhanced relationship with music	2	3		7	2	14	4
Trusting the power of music	10	1	3	1	4	19	5
Subtotal	12	4	3	8	6	33	
**PROFESSIONAL SIGNIFICANCE**							
Awareness of countertransference issues^*^	9	4	1		15	29	4
**Shifts in Clinical Emphasis**							
Client centeredness	16	8	18	8	9	59	5
Holistic approach			3	1		4	2
Therapeutic intention	3					3	1
Promoting expression	3		3		2	8	3
Flexibility	2	3				5	2
Eclectic practice				1		1	1
Relational support	6		2	2	2	12	4
Trusting musical space	2					2	1
**Professional Growth**							
Enhanced confidence	6	4				10	2
Taking risks for patients	2					2	1
Subtotal	49	19	27	12	28	135	
Grand Total	76	37	70	50	57	290	

* *indicates codes obtained by asking targeted questions*.

According to the interviews, participants believed that their personal experience of cancer made them better therapists in one way or the other. However, everyone echoed that one does not have to have cancer in order to become a good therapist. “You can be a great therapist without having been through all the experiences they had gone through.” Then, what is the difference? Their advice for developing higher level of understanding, empathy and connection are:

“Really listen, check yourself at the door, go in and be there for that person, work with the whole person, whole family” “Connect with your own feelings…think of times when you were in pain…when you needed to share emotional issues.” “(Learn from) those times when you struggle the most…Look yourself squarely in the eye, hear what it is you need to work on, be honest with yourself and find a mentor or someone who's going to help you move and grow.”

If I put them together as one message, it is “Know yourself, face your issues, and apply them clinically. If you can't, find someone to help you.” As a matter of fact, they have shared the same message throughout their interviews. The essence of this statement is “expansion of awareness” and the biggest outcome of that is “empathy” which is coded as “client-centeredness” in my analysis.

“The imagination is the sine qua non of empathy” (Kohn, 1990, p. 134). This means empathy is based on “imagined situations” and therefore the “essence of empathy is to share in the feelings of those who are in situations that we have never been in” (Musolf, 2003, p. 449). For these music therapists, they did not need to imagine to identify and empathize with the patients. It was not imagined empathy, but a lived empathy. They stated “really knowing what it's like, and what their needs are.” However, in these statements they are saying that they also learned to use this experience of “really knowing what it's like to be there” to inform and help them to empathize with other situations that they have not been in.

This expansion of awareness was evidenced in their self-awareness, and awareness of the whole client, music, time, environment, relationships, and their resources. Having this awareness doesn't make the problems and challenges go away, but they know what to do about them. Many of the participants said exactly the same thing “It's not about me, it's about them” however, the bigger message here is “In order to make it about them, know yourself well first.”

### Researcher's reflections on the participants

I would describe my experience of meeting and interviewing the therapists as loving, caring, kind, empathic, generous, humble, thoughtful, compassionate, courageous, strong, insightful, deep, driven, confident, warm people. They were eager and very willing to take time out of their busy life, invite me into their homes or offices, offer food or refreshments, and share incredible experiences, and the most personal stories with me. These encounters alone were highly enriching and therapeutic for me.

One very intriguing thing was that despite some of the similarities, they had beautifully idiosyncratic ways of thinking, coping with cancer, and working as therapists. I believe such individually unique stories helped me to describe the phenomena in full. At the time of the interviews, I was not aware of the shared core values because of the differences in participants. For example, even though the essence of their clinical work was “self-awareness,” what each participant needed to be aware of in order to cope and work effectively, was unique and different for each individual. Also, awareness was continuously evolving as one therapist said “I'm still learning from that experience. I'm not finished. I'm still surviving.” I wonder if this “remembering oneself as a cancer patient: maintaining that sense of identity and perhaps having those sustained worries about possible relapse” is something that keeps this self-awareness vital and powerful in their life.

### Limitations and recommendations for future studies

One thing to keep in mind is that research findings from a qualitative, phenomenologically-oriented study such as this, are not usually generalizable or applicable to other contexts. McLeod (2011) asserts that phenomenology strives to generate an “exhaustive description” of the phenomenon that leads to an understanding of the “essential structure” of the lived experience. In addition, this study had a small sample size of five which posed rather homogenous characteristics as all of them were female and most of them were white Caucasian, breast cancer survivors, and therapists who were trained in a cognitive behavioral orientation. Thus, future studies need to obtain participants with diverse backgrounds if possible.

Because this was the first study conducted on this topic, I set the scope of the study broadly encompassing all aspects of surviving cancer and returning to work as clinicians. As the first exploratory study, such a wide outlook on this phenomenon seemed more appropriate to generate an exhaustive description. Future studies may need to center on specific topics such as countertransference, bridging relationships between patients and medical staff, self-disclosure, expansion of awareness, specific clinical approaches and added modalities and dimensions in practice experienced by cancer surviving music therapists.

## Conclusion

In this study, five music therapists have shared their stories of surviving cancer. Cancer was painful in many aspects but they found ways to cope. It was stressful but they applied their own skills as therapists to remain in control. Cancer tested their relationships, but each therapist was able to mend those relationships into even healthier ones. Cancer took away many things that the participants initially considered valuable in their lives, but they found new meanings and discovered new opportunities from the experience. They became more fully aware of themselves, their patients, music and others. They were not just surviving cancer but thriving from it, and this transformative experience affected their perspectives on self, life, music, therapy and their clients.

This expanded awareness, and new way of living and working are something these five music therapists were willing to share so that other therapists, even if not affected by cancer, can begin to develop a greater appreciation for music in treatment, holistic care, self-awareness, and empathic care in order to address the needs of their clients to the fullest level possible.

## Author contributions

JL is the only author and researcher for this manuscript as he designed the study, interviewed the participants, analyzed data and devised the report.

### Conflict of interest statement

The author declares that the research was conducted in the absence of any commercial or financial relationships that could be construed as a potential conflict of interest.
